# Probing electron-phonon excitations in molecular junctions by quantum interference

**DOI:** 10.1038/srep20899

**Published:** 2016-02-11

**Authors:** C. Bessis, M. L. Della Rocca, C. Barraud, P. Martin, J. C. Lacroix, T. Markussen, P. Lafarge

**Affiliations:** 1 Université Paris Diderot, Sorbonne Paris Cité, MPQ, UMR 7162, CNRS, 75205 Paris Cedex 13, France; 2 Université Paris Diderot, Sorbonne Paris Cité, ITODYS, UMR 7086, CNRS, 15 rue J.-A. de Baïf, 75205 Paris Cedex 13, France; 3 QuantumWise A/S, Fruebjergvej 3, Box 4, DK-2100 Copenhagen, Denmark

## Abstract

Electron-phonon coupling is a fundamental inelastic interaction in condensed matter and in molecules. Here we probe phonon excitations using quantum interference in electron transport occurring in short chains of anthraquinone based molecular junctions. By studying the dependence of molecular junction’s conductance as a function of bias voltage and temperature, we show that inelastic scattering of electrons by phonons can be detected as features in conductance resulting from quenching of quantum interference. Our results are in agreement with density functional theory calculations and are well described by a generic two-site model in the framework of non-equilibrium Green’s functions formalism. The importance of the observed inelastic contribution to the current opens up new ways for exploring coherent electron transport through molecular devices.

Interaction between electrons and phonons (el-ph) is an ubiquitous process with fundamental as well as practical importance in systems ranging from superconductors to power dissipation in electronic devices. Molecular junctions are ideal test systems for studying el-ph interaction, since the influence from individual vibrational modes with well-defined energies can be observed in the current running through the device. However the inelastic signal from el-ph interaction is usually small compared to the elastic (non-interacting) contribution to the current. In certain molecules, the elastic current is dramatically suppressed due to destructive quantum interference, as recently demonstrated experimentally and theoretically for e.g. anthraquinone (AQ) based molecular junctions^1^,^2^,^3^,^4^,^5^,^6^,^7^,^8^,^9^. The quantum interference (QI) transport regime is achieved whenever two molecular orbitals coupled to a metal lead contribute simultaneously to charge transport, then multiple orbital pathways can interfere destructively^10^,^11^,^12^,^13^,^14^. In molecules showing QI effect, it has theoretically been predicted that the inelastic contribution to the current can be very significant and may even exceed the elastic part. While single-molecule measurements are challenging for molecules with QI effect since the current signal is close to the typical noise limit in such experiments^3^,^4^, junctions containing a layer of molecules do not suffer from this problem. Furthermore, a thorough study of the el-ph interaction requires the ability to control the temperature. Despite many theoretical works^15^,^16^,^17^,^18^ and one experimental result obtained in a slightly different regime^19^, the influence of electron-phonon interaction on quantum interference has not been clearly addressed experimentally. Besides, the experimental investigation of the low temperature and low energy behavior of quantum interference is lacking.

In this work we present measurements of short chains of anthraquinone based molecular layers, showing QI effect, embedded in solid-state devices. We realize highly controlled current and conductance measurements of such junctions in a large voltage range and with temperatures varying from 10 to 300 K. We are hereby able to use the QI effect in the AQ layer to study the el-ph interaction by measuring very large inelastic signals, in agreement with theoretical predictions^15^,^18^. Our experiments further show that the QI effect clearly remains visible even in the presence of el-ph interaction. Inelastic processes are revealed by the temperature dependence of the zero bias conductance and by the voltage dependence of conductance at low bias and low temperature. Here, we found that many phonon modes with characteristic energies ranging from 5 meV to 200 meV are activated in the molecular layer. Experimental data can be described by considering interacting quantum transport for electrons^15^,^20^,^21^. Data analysis is developed by using a generic two-site model^12^,^14^, supported also by DFT calculations. Within the model the electronic transmission function is calculated by the non-equilibrium Green function formalism (NEGF). Such approach captures the effect of quantum interference and allows an easy integration of the inelastic contribution to transport due to el-ph coupling^15^.

## Results

### Sample and measurement set-up

**Figure 1:**
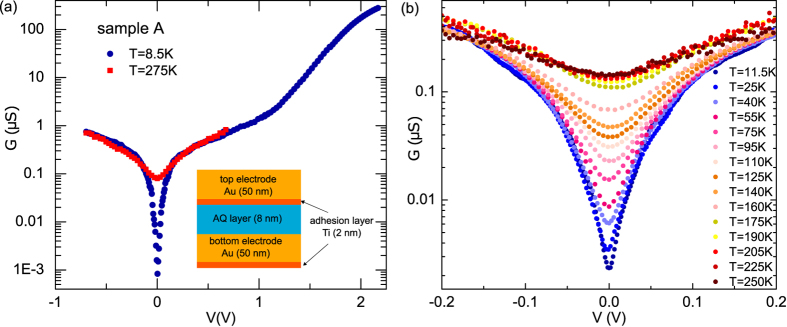
Voltage and temperature dependence of the junction conductance. (**a**) Measured conductance 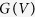 of an AQ-based junction with an area of 50 × 50 *μ*m^2^ at 8.5 K (blue dots) and 275 K (red squares). Inset: schematic of an AQ-based junction with the AQ layer covalently grafted on the Au bottom electrode. (**b**) 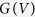 data for the same junction for temperatures ranging from 11.5 K to 250 K, in the voltage interval (−0.2 V, 0.2 V).

Junctions were made in a cross-bar geometry by embedding a ~8 nm-thick AQ grafted molecular layer between two metallic electrodes made of a Ti(2 nm)/Au(50 nm) bilayer^8^,^22^. Details on the sample fabrication and the electrochemical grafting of the AQ molecular layer are given in the Methods section. A sketch of an AQ-based junction with the AQ layer covalently grafted on the Au bottom electrode is given in the inset of Fig. 1(a). Even though the exact number of AQ units forming chains in the junction is unknown, an upper limit can be estimated by means of AFM thickness measurements. We found an AQ layer thickness of ~8 nm and by considering a typical dimension of ~1 nm for the single molecule, this implies a molecular layer containing at least 8 units along the chains. Samples were characterized by measuring simultaneously the current *I* and the differential conductance 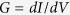 as a function of the applied voltage *V* using a low noise current amplifier and a standard homodyne detection at different temperatures ranging from 8.5 K to 275 K. A two probes measurement setup is used, the junction resistance (~MΩ) being much higher than the characteristic impedance of the connection lines (~50Ω). We present here results obtained on three different samples, hereafter referred to as A, B and C.

### Conductance of molecular junctions

**Figure 2:**
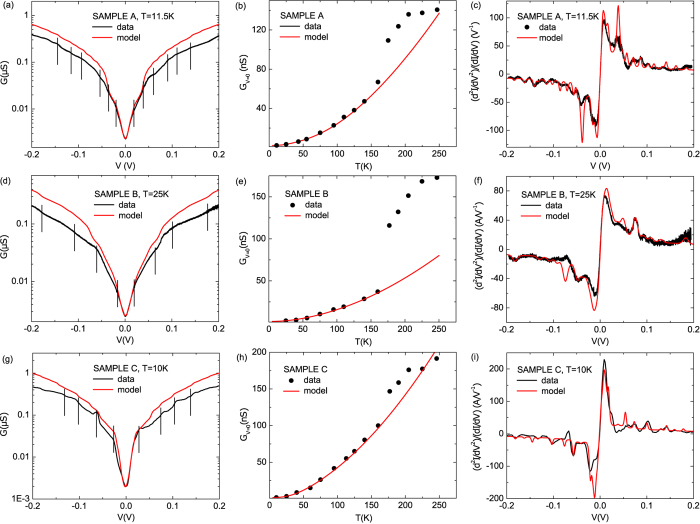
Data analysis of the voltage and temperature conductance dependences in the framework of the two-site model including el-ph interaction. (left) Measured conductance of sample A (**a**), B (**d**) and C (**g**) (black lines) at 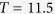 K, 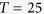 K and 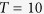 K respectively. Vertical lines indicate conductance features symmetrically distributed around zero voltage. (center) Temperature dependence of the zero bias conductance 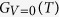 for sample A (**b**), B (**e**) and C (**h**) (black dots). A conductance jump is always present at 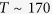 K, as a consequence we discard data for 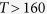 K. (right) Numerically calculated 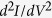 normalized to 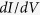 for sample A (**c**), B (**f**) and C (**i**) (black lines) emphasizing the conductance features as extremely marked peaks. Data in panels (**a**,**b**) for sample A, (**d**,**e**) for sample B and (**g**,**h**) for sample C are fitted simultaneously in the framework of the two-site model including el-ph interaction (solid red line).

Figure 1(a) shows the conductance of sample A in a large voltage range at 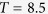 K (blue dots) and 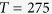 K (red squares). Systematically at ±2 V junctions become short circuits. The curve at 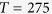 K reveals that temperature affects only the low voltage conductance. In the whole explored voltage range at low temperature the conductance changes remarkably by more than five orders of magnitude, showing a pronounced anti-resonance at low bias, a monotonic increase throughout the voltage range and a broad resonance at higher voltages. The zero bias anti-resonance is the characteristic signature of destructive quantum interference expected for AQ molecules^8^. Figure 1(b) shows the strong temperature dependence of the low voltage conductance for temperatures between 11.5 K and 250 K. This behavior is not consistent with charge transport mechanism based on quantum tunneling as described by e.g. the Simmons model^23^. Approaching room temperature the zero bias anomaly is reduced but still visible. This is the characteristic trend of all the analyzed samples. In Fig. 2(a,d,g), the 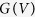 curve (black lines) is shown for the three samples at the lowest measured temperature in the voltage range (−0.2 V, 0.2 V). Conductance features distributed around zero voltage (as indicated by the vertical arrows) are clearly visible. In the following we show that these features are due to electron-phonon interaction and that the temperature dependence of the conductance originates from a combination of the broadening of the Fermi Dirac distribution functions and thermal excitations of vibrational degrees of freedom. Conductance features are better emphasized by the second derivative of the current with respect to the voltage 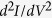 which is numerically calculated and plotted as a function of the applied voltage in Fig. 2(c,f,i) (black lines) after renormalization to 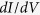. The conductance features appear as well defined peaks. The temperature dependence of the zero bias conductance is plotted in Fig. 2(b,e,h) (black dots) for the three samples. Note that a conductance jump is present at 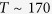 K on all the samples and can be related to thermally activated structural conformational changes^24^. The conductance jump for sample A is visible also on Fig. 1(b). We note that, even though a structural change has occurred in the molecular layer, the general shape of the curve keeps showing a well-defined minimum even at 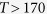 K, revealing further the robustness of the QI effect. Anyway for data analysis we focus on experimental measurements realized at 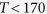 K, where the 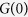 vs *T* curves show an almost parabolic behavior.

### Calculation of the electronic transmission function for an AQ chain

**Figure 3:**
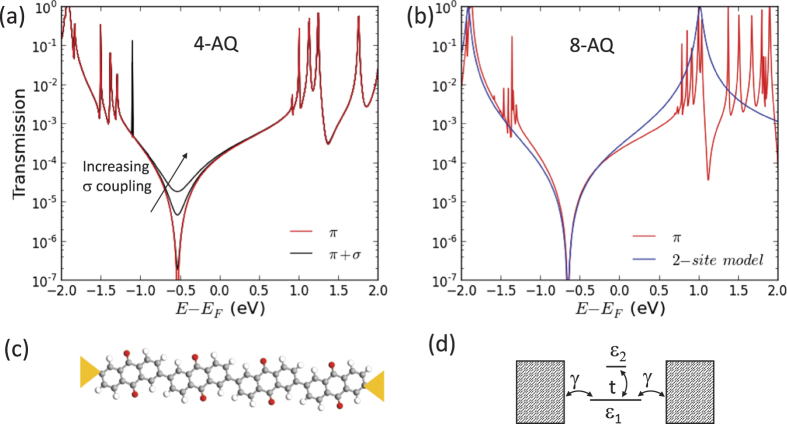
DFT transmission function for AQ based molecular chains. DFT calculated transmission function for a 4-AQ chain (**a**) and a 8-AQ chain (**b**) by considering only *π* states contribution (solid red curves) and 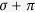 contribution (black curves) with different *σ* coupling to the electrodes. The molecules are coupled to wide-band electrodes at the sites indicated in panel (**c**) in the 4-AQ case. In panel (**b**) we also show the transmission function for a simple two-site model illustrated in panel (**d**) with parameters fitted to reproduce the DFT result. Close to the transmission dip, the DFT transmission is well reproduced by a two-site model.

To better understand quantum interference in AQ layer, we perform density functional theory (DFT) calculations on AQ chains. Figure 3 shows the transmission function calculated for chains of four AQ molecules (Fig. 3(a)) and eight AQ molecules (Fig. 3(b)). We model the electrode-molecule coupling using a wide-band approximation. This further allows us to differentiate between *π* and *σ*-contributions to the transport. Transport calculations using a fully atomistic description of the electrodes support the use of the simple wide-band model (see Supplementary Information).

We calculate the Hamiltonian and overlap matrices for an isolated molecule using the ATK package^25^. When considering only electrode coupling to *π*-type states (red curves), the transmission function shows a clear reduction over several orders of magnitude, signature of the destructive QI effect. By adding the *σ*-states (black curves in Fig. 3(a)), such transmission suppression becomes less pronounced for increasing electrode-*σ* coupling but the QI effect remains evident. Importantly, we find that the transmission function (for pure *π* coupling) for a chain of 4-AQs (a) is essentially the same as the transmission obtained for a 8-AQ chain (b). Similar correspondence is found when the *σ* coupling is included. This indicates that the electron transport across the AQ layer is relatively insensitive to the exact number of AQ molecules in the chain.

The simplest model capturing the quantum interference effect in molecular junctions is a “two-site” model introduced in refs 12,14. In this model (Fig. 3(d)), site 1 with energy 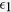 is coupled with a coupling constant *γ* to the right and left electrodes, while site 2 of energy 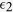 is fully decoupled from the electrodes and coupled by a coupling constant *t* to the first site. The model well reproduces a node in the electronic transmission function^12^ but also well describes a parabolic behavior of the zero bias conductance as a function of temperature^15^, as we observe.

In Fig. 3(b) we plot together with the DFT result for a 8-AQ chain, the transmission from a two-site model with parameters 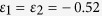 eV, 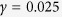 eV and 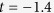 eV. In an energy range of ±0.4 eV around the QI transmission dip, the DFT result is well reproduced. This good agreement supports the use of a two-site model for describing charge transport through molecular chains with more than one AQ unit. We cannot rigorously extend the validity of the model to our molecular junctions. Anyway the presented calculations support its use as an empirical model able to reproduce the characteristic signature of QI effect, namely the electronic transmission function suppression, and to include easily the contribution due to el-ph interaction. In the following we present a quantitative analysis of our experimental data by using this theoretical description and we include the contribution of el-ph interaction to the conductance following ref. 15. Finally, to take into account the large area of our junctions the calculated conductance is multiplied by a scaling factor.

### Two site model and el-ph interaction

The total current for an interacting electron system is calculated using the Meir-Wingreen formula^20^. We consider a symmetric junction described in the framework of a two-site model and we apply a lowest order expansion (LOE)^15^,^21^. In LOE, the current is expressed as 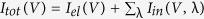, where 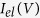 is the purely coherent elastic term given by the Landauer-Büttiker formula^26^ and 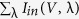 is the sum over the inelastic contributions related to each phonon mode indexed by λ with energy 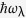
21. The term 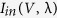 is given by


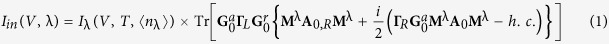


where


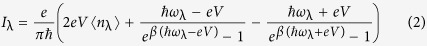


with 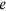 the elementary charge and 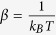, with 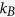 the Boltzmann constant. Here 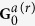 refers to the advanced (retarded) Green function without el-ph coupling, 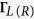 is the left (right) coupling matrix, 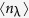 is the Bose-Einstein distribution for the phonon mode λ, 
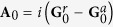
 and 
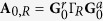
. Finally 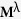 is the el-ph coupling matrix with elements 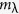. The LOE is valid when electronic Green’s functions vary slowly around the Fermi energy 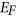 on the energy scale of the phonon energy.

Each time the voltage 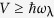, an incoming electron can scatter inelastically by emitting a phonon and a new inelastic transport channel is opened leading to a step in the conductance. The amplitude of the conductance steps depends on the el-ph coupling of the particular excited phonon mode. At higher temperature steps are smeared out. The zero bias conductance is also affected by the el-ph interaction especially at higher temperature where electrons are thermally excited. In the model calculations, the inelastic contribution to the current can be larger than the elastic one in particular close to the conductance steps.

On the basis of the described approach we fit simultaneously the 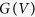 data at the lowest measured temperature and the 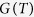 data at 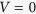 to a two-site model with interaction. Results for the three samples are shown as solid red lines in Fig. 2. In the two-site model the destructive interference anti-resonance occurs at 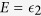, moreover the overall shape of the transmission function depends on the difference 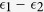 regardless of the anti-resonance energy position. The *t* and *γ* parameters affect the shape of the conductance curve acting mostly on the width of the anti-resonance in the transmission function. The phonon excitation modes 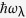 introduced in Eq. (1, 2) generate steps in the conductance curve and also contribute to the conductance shape at low voltage. As a consequence we set 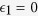 and for each sample we find the best fitting values for 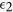, *t* and *γ* and for the set 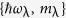 of phonon energy modes and couplings. The best description of data is obtained for sample A (B,C) with 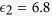 meV (8 meV, 4.7 meV), *t* = −2.4 eV (−2.4 eV, −2.5 eV) and *γ* = 0.4 eV (0.4 eV, 0.5 eV) and scaling factors of 125, 72 and 158 respectively. On average we introduce phonon modes in the energy range 5 meV–200 meV for each sample. A complete list of mode energies and respective couplings for the three samples is given in the Supplementary table S1. Even though the extracted phonon excitation energies vary from sample to sample, we find remarkably common values validating the reproducibility of our experimental findings and indicating that we are probing inelastic transport inside the molecular layer. These common modes are summarized in Table 1.

The 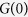 vs. *T* data are nicely reproduced in the temperature range of interest as well as the 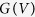 experimental curves at low voltage, however the model overestimates the conductance at higher voltages 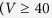 meV). This is expected since the theoretical description is valid for energies close to 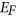. Furthermore, the theoretical model gives a good description of the peaks in the normalized 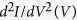 plot of Fig. 2(c,f,i) (solid red curve).

**Figure 4:**
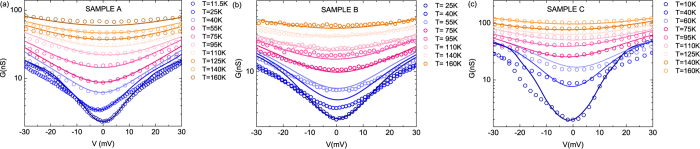
Temperature effect on the low voltage conductance. Low bias conductance data 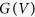 (open dots) for sample A (**a**), B (**b**) and C (**c**) for temperature ranging from 10 K to 160 K compared to the theoretical description (solid lines) combining elastic and inelastic contribution to the current. Curves are reproduced by using the parameters extracted from the fitting procedure described in Fig. 2 and by considering the different temperatures at which data are recorded.

The quality of the fit at low voltage is confirmed by the very good agreement between experiment and theory at higher temperatures as shown in Fig. 4. For each sample we use the fitting parameters extracted at the lowest temperature to compute the 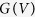 dependence for higher temperatures up to 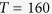 K. Figure 4 shows the experimental 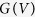 curves (open dots) in the low voltage range (−30 meV, 30 meV) at different temperatures (10 K−160 K) together with the calculated conductance (solid lines). The effect of temperature is included in the coherent contribution to the current via the electrodes Fermi functions and also in the variation of 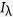 given in Eq. (2). Note that no adjusting parameter is required in this comparison.

## Discussion

The common phonon modes listed in Table 1 are not evenly spaced in energy. However we can not distinguish to which extent such modes are independent or if we are able to measure higher order harmonics of few fundamental modes, which are in principle visible in QI based junctions^18^. DFT calculations performed on AQ chain containing 3 units (see Supplementary Information) show that low-energy phonon modes exist for such a molecular structure, that couple strongly to the frontier molecular orbitals. The phonon mode which seems to dominate consists in out-of-plane vibrations and has a characteristic energy of ≈5 meV. Remarkably infrared spectra of AQ single crystals and solutions show vibrational modes with energies very close to our findings, with peaks at 4 meV (35 cm^−1^), 10 meV (83 cm^−1^), 21 meV (167 cm^−1^), 51 meV (408 cm^−1^), 60 meV (486 cm^−1^), 77 meV (620 cm^−1^), 102 meV (820 cm^−1^), 110 meV (885 cm^−1^), 131 meV (1054 cm^−1^), 180 meV (1454 cm^−1^) and 183 meV (1474 cm^−1^)^27^. Moreover note that well known vibrational energies can be recognized^28^, such as the Au-C bond stretching mode with an energy of ~50 meV^29^ as well as the C-C bond stretching with energy ~180 meV^30^. Inelastic signals at these energies are also clearly seen in the DFT calculated IETS (see Supplementary Information). Vibration excitation spectra in the same energy range has been recently reported also for Alq_3_ based molecular junctions with a similar cross-bar geometry^31^.

In conclusion, we have demonstrated that quantum interference effect can probe phonon excitations in short chains of anthraquinone based planar junctions. We show that the opening of inelastic transport channels by phonon excitations is visible in the conductance of the molecular junctions due to the reduction of the elastic current by quantum interference. A quantitative analysis based on a two-site model allows to unravel clear signatures of el-ph interaction in electronic transport whose energies are consistent with previously reported values. The dominant role of inelastic contributions to the current opens the way towards new spectroscopic methods for molecular conductors taking advantage of the coherent transport of electrons and towards molecular devices driven by external inelastic signals such as photonic excitations.

## Methods

### Sample fabrication

Junctions were made using conventional micro-fabrication techniques in cross-bar geometry^8^,^22^. Junction area is of the order of 50 × 50 *μ*m^2^. The bottom and top electrodes are made of a 2 nm Ti/50 nm Au bilayer. The Ti layer improves Au adhesion on the Si/SiO_2_ substrate and reduces Au diffusion through the molecular layer. The base electrode is obtained by UV-lithography followed by e-beam evaporation in a base pressure of ~10^−8^ mbar. The AQ layer is then grafted by the method of electroreduction of the corresponding diazonium salt^8^,^22^. The thickness of the grafted organic layer was estimated by atomic force microscopy (AFM) by comparing the profile of the Au bare electrode and the profile of the same electrode after AQ grafting. The resulting thickness is 8.0 ± 0.8 nm. This implies a molecular layer containing at least 8 units along the chain, consistent with measured surface concentration. The top Ti/Au electrode is finally gentle evaporated (rate ~0.02 nm/s) through a shadow mask on the freshly deposed molecular layer always in a base pressure of ~10^−8^ mbar. Such procedure makes sure that the molecular layer is not exposed to any chemical treatment after its grafting, preserving its quality and avoiding contaminations.

### Electroreduction of AQD

The electroreduction method adopted to graft the AQ layer on the base electrode of the molecular junctions^32^,^33^ ensures the formation of a robust, thin layer of molecules bonded covalently to the Au surface. Cyclic voltammetry (20th scan) is realized on the Au junction base electrode for the electroreduction of 9,10-dioxo-1-anthracenediazonium salt in 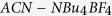 solution (5 × 10^−3^ M in ACN solution with 0.1 M of 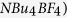. The scan rate is 100 mVs^−1^. After the first cycle characterized by an irreversible wave corresponding to the reduction of diazonium salt, the subsequent scans do not exhibit this behavior indicating the covalent grafting of gold with other aryl diazonium compounds^34^,^35^. As AQ is electroactive (reduction to the hydroquinone group^36^,^37^), the surface concentration (*G*) of AQ was determined by the charge integration under the cyclic voltammetry (CV) peaks 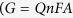, where *Q* is the amount of charge consumed, *n* is the number of electron involved 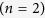, *F* is the faraday constant and *A* is the area of electrode). The estimated surface coverage of AQ was around 1.5 × 10^−9^ mol cm^−2^ for 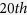 cycles of grafting and it corresponds to more than one monolayer. The uniformity of the AQ grafted layer was evaluated by testing the blocking effect of AQ modified electrode towards the *Fe(CN)*_6_^3-^/^4-^ redox couple by cyclic voltammetry. For an effective electron transfer process, *Fe(CN)*_6_^3-^ ions should interact with gold electrode^34^, this does not occur when a film completely covers the substrate surface. The CV analysis of the bare and modified electrodes in 5 mM *K*_3_[*Fe(CN)*_6_] solution shows that for 20th cycles the CV response of redox probe is completely suppressed.

## Additional Information

**How to cite this article**: Bessis, C. *et al*. Probing electron-phonon excitations in molecular junctions by quantum interference. *Sci. Rep*. **6**, 20899; doi: 10.1038/srep20899 (2016).

## Supplementary Material

Supplementary Information

## Figures and Tables

**Table 1:** Comparison of the el-ph modes energies extracted from the analysis of transport properties of samples A, B and C.

Sample A	Sample B	Sample C
6 ± 1	5 ± 1	5 ± 2
10 ± 1	10 ± 2	10 ± 2
15 ± 2	15 ± 2	15 ± 1
18 ± 2	17 ± 2	20 ± 1
40 ± 2	37 ± 3	40 ± 2
57.5 ± 3	51 ± 3	56 ± 2
71 ± 3	76 ± 2	75 ± 4
102.5 ± 3	109 ± 4	102 ± 3
133 ± 2	130 ± 4	132 ± 3
184 ± 2	183 ± 4	180 ± 4

Energies are expressed in meV.

## References

[b1] Fracasso D., Valkenier H., Hummelen J. C., Solomon G. C. & Chiechi R. C. Evidence for Quantum Interference in SAMs of Arylethynylene Thiolates in Tunneling Junctions with Eutectic Ga–In (EGaIn) Top-Contacts. J. Am. Chem. Soc. 133, 9556 (2011). 21561156 10.1021/ja202471m

[b2] Guédon C. M., Valkenier H., Markussen T., Thygesen K. S., Hummelen J. C. & van der Molen S. J. Observation of quantum interference in molecular charge transport. Nat. Nanotechnol. 7, 305 (2012). 22447160 10.1038/nnano.2012.37

[b3] Hong W., Valkenier H., Mészáros G., Manrique D. Z., Mishchenko A., Putz A., García P. M., Lambert C. J., Hummelen J. C. & Wandlowski T. An MCBJ case study: The influence of -conjugation on the single-molecule conductance at a solid/liquid interface. Belstein J. Nanotechnol. 2, 699 (2011). 10.3762/bjnano.2.76 PMC3201624 22043460

[b4] Aradhya S. V., Meisner J. S., Krikorian M., Ahn S., Parmeswaran R., Steigerwald M. L., Nuckolls C. & Venkataraman L. Dissecting Contact Mechanics from Quantum Interference in Single-Molecule Junctions of Stilbene Derivatives. Nano Lett. 12, 1643 (2012). 10.1021/nl2045815 22352939

[b5] Valkenier H., Guédon C. M., Markussen T., Thygesen K. S., van der Molen S. J. & Hummelen J. C. Cross-conjugation and quantum interference: a general correlation? Phys. Chem. Chem. Phys. 16, 653 (2014). 24270575 10.1039/c3cp53866d

[b6] Darwish N., Díez-Pérez I., Da Silva P., Tao N., Gooding J. J. & Paddon-Row M. N. Observation of Electrochemically Controlled Quantum Interference in a Single Anthraquinone-Based Norbornylogous Bridge Molecule. Angew. Chem. Int. Ed. 51, 3203 (2012). 10.1002/anie.201107765 22334514

[b7] Arroyo C. R., Tarkuc S., Frisenda R., Seldenthuis J. S., Woerde C. H. M., Eelkema R., Grozema F. C. & van der Zant H. S. J. Signatures of Quantum Interference Effects on Charge Transport Through a Single Benzene Ring. Angew. Chem. Int. Ed. 526, 3152 (2013). 10.1002/anie.201207667 23386366

[b8] Rabache V., Chaste J., Petit P., Della Rocca M. L., Martin P., Lacroix J. C., McCreery R. L. & Lafarge P. Direct Observation of Large Quantum Interference Effect in Anthraquinone Solid-State Junctions. J. Am. Chem. Soc. 135, 10218 (2013). 23805821 10.1021/ja403577u

[b9] Koole M., Thijssen J. M., Valkenier H., Hummelen J. C. & van der Zant H. S. J. Electric-Field Control of Interfering Transport Pathways in a Single-Molecule Anthraquinone Transistor. Nano Lett. 15, 5569 (2015). 26182342 10.1021/acs.nanolett.5b02188

[b10] Cardamone D. M., Stafford C. A. & Mazumdar S. Controlling Quantum Transport through a Single Molecule. Nano Lett. 6, 2422 (2006). 17090067 10.1021/nl0608442

[b11] Stadler R., Ami S., Forshaw M. & Joachim C. Integrating logic functions inside a single molecule. Nanotechnology 15, S115 (2004).

[b12] Solomon G. C., Andrews D. Q., Hansen T., Goldsmith R. H., Wasielewski M. R., Van Duyne R. P. & Ratner M. A. Understanding quantum interference in coherent molecular conduction. J. Chem. Phys. 129, 054701 (2008). 18698915 10.1063/1.2958275

[b13] Darau D., Begemann G., Donarini A. & Grifoni M. Interference effects on the transport characteristics of a benzene single-electron transistor. Phys. Rev. B 79, 235404 (2009).

[b14] Markussen T., Schiötz J. & Thygesen K. S. Electrochemical control of quantum interference in anthraquinone-based molecular switches. J. Chem. Phys. 132, 224104 (2010). 20550387 10.1063/1.3451265

[b15] Markussen T. & Thygesen K. S. Temperature effects on quantum interference in molecular junctions. Phys. Rev. B 89, 085420 (2014).

[b16] Härtle R., Butzin M., Rubio-Pons O. & Thoss M. Quantum Interference and Decoherence in Single-Molecule Junctions: How Vibrations Induce Electrical Current. Phys. Rev. Lett. 107, 046802 (2011). 21867029 10.1103/PhysRevLett.107.046802

[b17] Härtle R., Butzin M. & Thoss M. Vibrationally induced decoherence in single-molecule junctions. Phys. Rev. B 87, 085422 (2013).

[b18] Lykkebo J., Gagliardi A., Pecchia A. & Solomon G. C. Strong Overtones Modes in Inelastic Electron Tunneling Spectroscopy with Cross-Conjugated Molecules: A Prediction from Theory. ACS Nano 7, 9183 (2013). 24067128 10.1021/nn4037915 PMC3807527

[b19] Ballmann S., Härtle R., Coto P. B., Elbing M., Mayor M., Bryce M. R., Thoss M. & Weber H. B. Experimental Evidence for Quantum Interference and Vibrationally Induced Decoherence in Single-Molecule Junctions. Phys. Rev. Lett. 109, 056801 (2012). 23006194 10.1103/PhysRevLett.109.056801

[b20] Meir Y. & Wingreen N. S. Landauer formula for the current through an interacting electron region. Phys. Rev. Lett. 68, 2512 (1992). 10045416 10.1103/PhysRevLett.68.2512

[b21] Frederiksen T., Paulsson M., Brandbyge M. & Jauho A.-P. Inelastic transport theory from first principles: Methodology and application to nanoscale devices. Phys. Rev. B 75, 205413 (2007).

[b22] Martin P., Della Rocca M. L., Lafarge P. & Lacroix J. C. Organic Electrodes Based on Grafted Oligothiophene Units in Ultrathin, Large-Area Molecular Junctions. J. Am. Chem. Soc. 134, 154 (2012). 22148633 10.1021/ja209914d

[b23] Simmons J. G. Generalized Formula for the Electric Tunnel Effect between Similar Electrodes Separated by a Thin Insulating Film. J. Appl. Phys. 34, 1793 (1963).

[b24] Yan H., Bergren A. J. & McCreery R. L. All-Carbon Molecular Tunnel Junctions. J. Appl. Chem. Soc. 133, 19168 (2011). 10.1021/ja206619a 22017204

[b25] Atomistix ToolKit version 2015, QuantumWise A/S (www.quantumwise.com)

[b26] Blanter Y. M. & Büttiker M. Shot noise in mesoscopic conductors. Phys. Rep. 336, 1 (2000).

[b27] Pecile C. & Lunelli B. Polarized Infrared Spectra of Single Crystals of 9,10-Anthraquinone and 9,10-Anthraquinone-d_8_. J. Chem. Phys. 46, 2109 (1967).

[b28] Socrates G. Infrared and Raman Characteristic Group Frequencies: Tables and Charts (John Wiley & Sons, 2004).

[b29] Laurentius L., Stoyanov S. R., Gusarov S., Kovalenko A., Du R., Lopinski G. P. & McDermott M. T. Diazonium-Derived Aryl Films on Gold Nanoparticles: Evidence for a Carbon–Gold Covalent Bond. ACS Nano 5, 4219 (2011). 21520960 10.1021/nn201110r

[b30] Ricci A., Bonazzola C. & Calvo E. An FT-IRRAS study of nitrophenyl mono- and multilayers electro-deposited on gold by reduction of the diazonium salt. J. Phys. Chem. Chem. Phys. 8, 4297 (2006). 10.1039/b609497j 16986072

[b31] Galbiati M., Tatay S., Delprat S., Khanh H. L., Servet B., Deranlot C., Collin S., Seneor P., Mattana R. & Petroff F. Is spin transport through molecules really occurring in organic spin valves? A combined magnetoresistance and inelastic electron tunnelling spectroscopy study. Appl. Phys. Lett. 106, 082408 (2015).

[b32] Bousquet A., Ceccato M., Hinge M., Pedersen S. U. & Daasbjerg K. Redox Grafting of Diazotated Anthraquinone as a Means of Forming Thick Conducting Organic Films. Langmuir 28, 1267 (2012). 22175534 10.1021/la203657n

[b33] Mesnage A., Lefèvre X., Jégou P., Deniau G. & Palacin S. Spontaneous Grafting of Diazonium Salts: Chemical Mechanism on Metallic Surfaces. Langmuir 28, 11767 (2012). 22793962 10.1021/la3011103

[b34] Chen P. & McCreery R. L. Control of Electron Transfer Kinetics at Glassy Carbon Electrodes by Specific Surface Modification. Anal. Chem. 68, 3958 (1996).

[b35] Bélanger D. & Pinson J. Electrografting: a powerful method for surface modification. Chem. Soc. Rev. 40, 3995 (2011). 21503288 10.1039/c0cs00149j

[b36] Reilson R., Kullapere M. & Tammeveski K. Blocking Behavior of Covalently Attached Anthraquinone Towards Solution-Based Redox Probes. Electroanalysis 22, 513 (2010).

[b37] Kullapere M., Marandi M., Sammelselg V., Menezes H. A., Maia G. & Tammeveski K. Surface modification of gold electrodes with anthraquinone diazonium cations. Electrochem. commun. 11, 405 (2009).

