# Supplementary material for: Probing electron-phonon excitations in molecular junctions by quantum interference
Source: Sci Rep. 2016 Feb 11;6:20899. doi: 10.1038/srep20899 (PMC4750039; doi:10.1038/srep20899)
Supplement: Supplementary Information [file srep20899-s1.pdf]

Probing electron-phonon excitations in molecular junctions by quantum interference

Supplementary information

C. Bessis,[1] M. L. Della Rocca,[1,*] C. Barraud,[1] P. Martin,[2] J. C. Lacroix,[2] T. Markussen,[3] and P. Lafarge[1]

[1]Université Paris Diderot, Sorbonne Paris Cité, MPQ, UMR 7162, CNRS, 75205 Paris Cedex 13, France

[2]Université Paris Diderot, Sorbonne Paris Cité, ITODYS, UMR 7086, CNRS, 15 rue J.-A. de Baïf, 75205 Paris Cedex 13, France

[3]QuantumWise A/S, Fruebjergvej 3, Box 4, DK-2100 Copenhagen, Denmark

[*]Electronic address: `maria-luisa.della-rocca@univ-paris-diderot.fr`

DFT CALCULATION OF THE TRANSMISSION FUNCTION FOR AQ MOLECULAR CHAINS

In all of the DFT-based calculations we have used the ATK package [3]. We adopt the generalized gradient approximation (GGA) for the exchange-correlation functional using the Perdew-Burke-Ernzerhof (PBE) functional [5]. We apply a basis of localized atomic orbitals with double-zeta plus polarization orbitals.

Transmission through molecules coupled to wide-band electrodes

We calculate the transmission function through chains of AQ molecules with a varying number of AQ units. Since we have no experimental knowledge of the binding geometry between the molecules and the Au electrodes, we present in the main paper simplified calculations of the transmission function through AQ molecules connected to artificial wide-band electrodes.

After a structural relaxation until the maximum force is less that 0.01 eV/$\mathring{A}$, we obtain from ATK the Hamiltonian **H** and overlap matrices **S** of free molecules, i.e. with no description of the electrodes. We then include the coupling to the electrodes through left- and right self-energy matrices, $\mathbf{\Sigma}_{L,R}$, where e.g. $\mathbf{\Sigma}_L$ only has non-zero elements on the diagonal indices corresponding to basis functions located on the carbon atom close to the left electrode. Although the molecules are passivated with hydrogen, we couple the wide-band electrodes to the closest carbon atom in order to probe the important contribution from the π-channels. Coupling to the hydrogen 1s-orbital does not couple with the π-system, but only with the σ-orbitals. We couple s, p_x, and p_y orbitals equally with a value of $-i\gamma_\sigma$ and the p_z orbital with a value of $-i\gamma_\pi$. The Fermi energy is chosen as midway between the HOMO and LUMO orbitals of the free molecules. The retarded Green's function is now obtained as

$$\mathbf{G}(E) = (E\mathbf{S} - \mathbf{H} - \mathbf{\Sigma}_L - \mathbf{\Sigma}_R)^{-1} \tag{1}$$

and the transmission is calculated with the usual NEGF trace formula

$$T(E) = \mathrm{Tr}[\mathbf{G}(E)\Gamma_L \mathbf{G}^\dagger(E)\Gamma_R]. \tag{2}$$

Besides its simplicity, this approach has the advantage to allow an easy examination of the contributions coming from the π and σ systems separately. The results of this analysis is shown in Fig. 3 in the main text.

Transmission through molecules with a fully atomistic description of electrodes

In addition to the transmission calculations through the free molecules we also performed transmission calculations for AQ-molecules coupled to Au electrodes [4]. The structure of the molecules in the junction has been relaxed until the forces are smaller than 0.05 eV/$\AA$. The final geometry is shown in Fig. S1 (a). At both ends, the molecule is passivated with hydrogen and binds to the Au (111) surface with an Au-H bond. The molecular configuration has been relaxed while keeping the Au atomic positions fixed.

The transverse unit cell in the DFT calculations including Au electrodes is $8.65\AA\times 9.99\AA$. This choise is motivated to essentially avoid interactions between periodically repeated molecules. Different packing densities could indeed lead to small energy shifts, but we believe the main conclusions are unaffected by the specific packing density. The unit cell length (in the transport direction) is $44.20\AA$ and was found by minimizing the forces, while fixing the internal coordinates of the Au atoms allowing only their center of mass to move in the relaxation.

In the self-consistent DFT calculation we use a transverse k-point sampling of 5×5.

Figure S1 (b) shows the transmission function for this junction geometry. In good agreement with the free molecule transmissions, we again observe a transmission dip at -0.5 eV below the Fermi energy. We note that the exact position of the dip as well as the transmission peaks are not expected to be accurately predicted with the semi-local GGA used in this work. However, we expect the trend to be valid.

IETS from DFT

In addition to the elastic DFT-based transmission calculations above, we have also performed calculations of the inelastic current for the junction structure shown in Fig. S1 (a). We calculated the vibrational modes of the molecule attached to Au electrodes, but disregard atomic displacements in the electrodes. The electron-phonon coupling as well as the inelastic current is calculated within DFT using the Lowest-Order-Expanssion (LOE) [2] as implemented in ATK-2015.

Figure S2 (a) shows the IETS spectra of the AQ3-junction. There are notable similarities with the experimentally obtained IETS spectra shown in Fig. S2 (b) (also shown in Fig. 2 in the main text). In particular the largest peak at a bias voltage of $\sim 5\,\mathrm{meV}$ is very similar in experiments and calculations. Also, the general order of magnitude of the inelastic signals

(a)

(b)

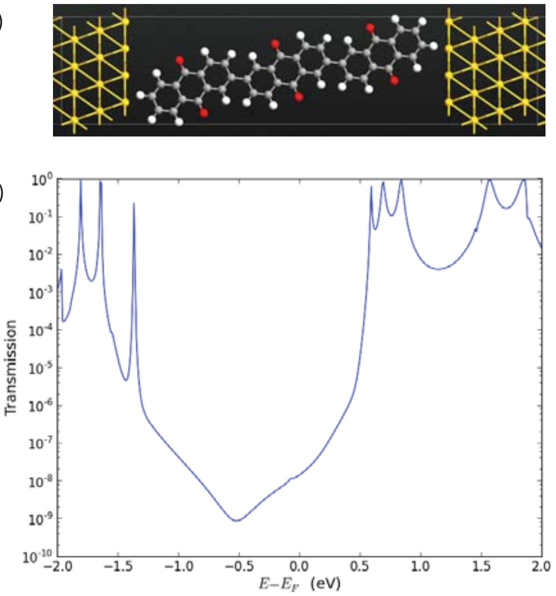

FIG. S1: **Transmission of an AQ molecular chain coupled to Au electrodes.** (a) Junction structure used for the DFT transport calculations. Molecules are coupled to Au electrodes via Au-H bonds. (b) Calculated transmission. A clear transmission dip is present at -0.5 eV below the Fermi energy in agreement with the free molecule calculation using wide-band electrodes (Fig. 3 in main text).

agree well between theory and experiments, thus supporting the interpretation that the signals in the experimental IETS spectra are due to electron-phonon interaction.

The phonon mode responsible for the largest peak at 5 meV is plotted in Fig. S3. The mode is essentially an out-of-plane vibration with a phonon energy of 5 meV. Looking closer at the mode one observes that the atomic displacements are particularly pronounced for the oxygen atoms.

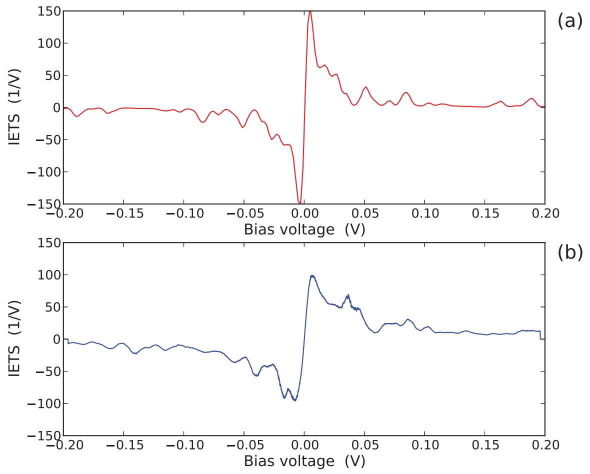

FIG. S2: **IETS spectra.** IETS spectra obtained from DFT-LOE (a) and from experiments (b). Both the position and magnitude of the main peak at 5 meV are the same. The overall shape as well as the magnitude of IETS peaks are also similar.

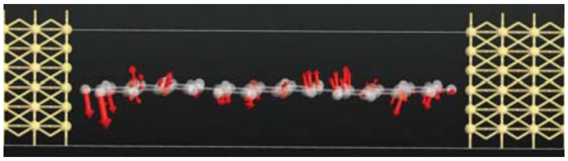

FIG. S3: **Low energy phonon mode.** Phonon mode responsible for the largest IETS peak. The mode is essentially an out-of-plane vibration with a phonon energy of 5 meV.

EXTRACTED ENERGIES OF THE el-ph MODES AND RESPECTIVE COUPLINGS

In the following Table S1 we summarize the el-ph energies and the respective coupling extracted from the fitting procedure of the three samples analyzed in the main text.

We can not know to which extent we are measuring independent modes or multiple phonon excitations.

TABLE S1: **Vibrational excited modes.** el-ph modes energies ($\hbar\omega_\lambda$) and respective couplings ($\frac{m_\lambda}{\hbar\omega_\lambda}$) used in the fitting procedure presented in the main text relative to sample A, B and C.

Sample A	
Ph energy $\hbar\omega_\lambda$ (meV)	coupling $\frac{m_\lambda}{\hbar\omega_\lambda}$
6	2
10	1.3
15	0.87
18	0.89
32	0.31
38	0.92
40	1
43	0.7
48	0.73
57.5	0.7
71	0.56
86	0.56
102.5	0.34
115	0.35
133	0.22
141	0.42
162	0.31
184	0.22

Sample B	
Ph energy $\hbar\omega_\lambda$ (meV)	coupling $\frac{m_\lambda}{\hbar\omega_\lambda}$
5	2
7.5	1.3
10	1
15	0.87
17	1.47
25	1
37	0.67
48	0.58
51	0.74
76	1.0
93	0.54
109	0.46
130	0.38
183	0.49

Sample C	
Ph energy $\hbar\omega_\lambda$ (meV)	coupling $\frac{m_\lambda}{\hbar\omega_\lambda}$
5	1.1
10	1.2
12	1.25
15	1.33
20	1.75
25	0.4
40	0.25
56	0.8
75	0.53
102	0.49
132	0.23
144	0.42
161	0.22
180	0.28

[1] Markussen, T. & Thygesen, K. S. Temperature effects on quantum interference in molecular junctions. *Phys. Rev. B* **89**, 085420, (2014).

[2] Frederiksen, T., Paulsson, M., Brandbyge M. & Jauho, A.-P. Inelastic transport theory from first principles: Methodology and application to nanoscale devices. *Phys. Rev. B* **75**, 205413

(2007).

[3] Atomistix ToolKit version 2015, QuantumWise A/S (www.quantumwise.com)

[4] Brandbyge, M., Mozos, J.-L., Ordejn, P., Taylor, J. & Stokbro, K. Density-functional method for nonequilibrium electron transport. *Phys. Rev. B* **65**, 165401 (2002)

[5] Perdew, J. P., Burke, K. & Ernzerhof, M. Generalized Gradient Approximation Made Simple. *Phys. Rev. Lett.* **77**, 3865, (1996).
